# Terlipressin combined with conservative fluid management attenuates hemorrhagic shock-induced acute kidney injury in rats

**DOI:** 10.1038/s41598-022-24982-0

**Published:** 2022-11-28

**Authors:** Leticia Urbano Cardoso Castro, Denise Aya Otsuki, Talita Rojas Sanches, Felipe Lima Souza, Mirela Aparecida Rodrigues Santinho, Cleonice da Silva, Irene de Lourdes Noronha, Amaro Nunes Duarte-Neto, Samirah Abreu Gomes, Luiz-Marcelo Sá Malbouisson, Lucia Andrade

**Affiliations:** 1 Laboratory of Basic Science in Renal Diseases, Division of Nephrology, University of São Paulo School of Medicine, Av. Dr. Arnaldo, 455, 3º Andar, sala 3310, São Paulo, SP CEP 01246-903 Brazil; 2 Laboratory of Anesthesiology, Division of Anesthesiology, University of São Paulo School of Medicine, São Paulo, Brazil; 3 Laboratory of Cellular, Genetic, and Molecular Nephrology, Renal Division, University of São Paulo School of Medicine, São Paulo, Brazil; 4 Department of Pathology, University of São Paulo School of Medicine, São Paulo, Brazil

**Keywords:** Kidney, Nephrology, Kidney, Nephrons

## Abstract

Hemorrhagic shock (HS), a major cause of trauma-related mortality, is mainly treated by crystalloid fluid administration, typically with lactated Ringer’s (LR). Despite beneficial hemodynamic effects, such as the restoration of mean arterial pressure (MAP), LR administration has major side effects, including organ damage due to edema. One strategy to avoid such effects is pre-hospitalization intravenous administration of the potent vasoconstrictor terlipressin, which can restore hemodynamic stability/homeostasis and has anti-inflammatory effects. Wistar rats were subjected to HS for 60 min, at a target MAP of 30–40 mmHg, thereafter being allocated to receive LR infusion at 3 times the volume of the blood withdrawn (liberal fluid management); at 2 times the volume (conservative fluid management), plus terlipressin (10 µg/100 g body weight); and at an equal volume (conservative fluid management), plus terlipressin (10 µg/100 g body weight). A control group comprised rats not subjected to HS and receiving no fluid resuscitation or treatment. At 15 min after fluid resuscitation/treatment, the blood previously withdrawn was reinfused. At 24 h after HS, MAP was higher among the terlipressin-treated animals. Terlipressin also improved post-HS survival and provided significant improvements in glomerular/tubular function (creatinine clearance), neutrophil gelatinase-associated lipocalin expression, fractional excretion of sodium, aquaporin 2 expression, tubular injury, macrophage infiltration, interleukin 6 levels, interleukin 18 levels, and nuclear factor kappa B expression. In terlipressin-treated animals, there was also significantly higher angiotensin II type 1 receptor expression and normalization of arginine vasopressin 1a receptor expression. Terlipressin associated with conservative fluid management could be a viable therapy for HS-induced acute kidney injury, likely attenuating such injury by modulating the inflammatory response via the arginine vasopressin 1a receptor.

## Introduction

Hemorrhagic shock (HS) accounts for 40% of deaths among trauma patients^1,2^. The main causes of hemorrhage are gastrointestinal bleeding, the rupture of blood vessels, including the abdominal aorta, and coagulopathies^3^. HS can be seen as global ischemia/reperfusion injury and triggers inflammatory responses in vessels and organs^4–7^. Blood loss can cause hypoperfusion and lead to multiple organ failure, the kidney being the organ most often affected; kidney failure increases mortality significantly^8^. Acute kidney injury (AKI) is one of the most common outcomes of severe hypovolemia. HS-induced AKI has a complex pathophysiology involving high release of systemic pro- and anti-inflammatory mediators, together with increased macrophage infiltration, as well as vasoconstriction, endothelial dysfunction, and induction of tubular cell necrosis^9,10^. Mayeur et al. demonstrated that even 21 days after HS, there was renal function impairment, which manifested as an increased serum cystatin C level, a higher histological score for renal injury, and an increase in the level of hypoxia-inducible factor 1 expression, which is indicative of tissue hypoxia^11^. Nuclear factor kappa B (NF-κB) is one of the pivotal elements in the triggering of an inflammatory response in AKI^12^.

The current standard resuscitation protocol for HS mandates the use of liberal fluid management, which involves the infusion of large volumes of crystalloid solutions such as lactated Ringer’s (LR). However, that practice can have adverse effects, such as interstitial edema in the gut, lungs, and kidneys^13–16^; increased production of pro-inflammatory cytokines^17^; and increased intracranial pressure^18^. Other studies have demonstrated additional adverse effects, including coagulopathies due to the large dilution, hypothermia related to LR administration at an inappropriate temperature, and changes in the serum pH^19,20^. It has long been known that, for intensive care unit patients with HS, a conservative fluid management strategy results in longer survival, a greater number of ventilator-free days, and shorter hospital stays than does a liberal fluid management strategy^21,22^. Early vasopressor use can restore hemodynamic parameters and organ perfusion, thus reducing the need for aggressive fluid therapy and avoiding fluid overload.

Terlipressin is a synthetic, long-acting analogue of arginine vasopressin (AVP). Terlipressin contains a peptide designated the natural hormone lysine-vasopressin, the innate AVP analogue in pigs. It acts through the AVP 1a receptor (V1aR), which has been detected in several regions of the kidney, including the thick ascending limb, collecting ducts, and renal vascular cells^23^, as well as in macula densa cells^24^. Terlipressin has been studied as a vasoactive drug in the management of catecholamine-resistant arterial hypotension in septic shock^15^, liver failure^25^, and acute gastrointestinal bleeding^26^. The effects of terlipressin consist of vasoconstrictor activity in vascular smooth muscle cells and pronounced vasoconstriction within the splanchnic circulation, such vasoconstriction having been shown to redistribute blood flow in order to restore perfusion pressure to organs such as the liver, kidneys, and brain^27,28^, as well as increasing survival. In two previous studies, both involving a porcine model of HS, our group demonstrated that the administration of terlipressin was effective in normalizing cerebral perfusion pressure, as well as cerebral markers of water balance, oxidative damage, and apoptosis^6^; we also showed that, at 120 min after HS, terlipressin continued to protect renal function, water transporters, sodium transporters, and kidney tissue^29^. A V1aR agonist might act as an immunomodulator to repress pro-inflammatory cytokine expression^30^.

The objective of the present study was to determine whether terlipressin combined with conservative fluid management protects against renal injury in HS-induced AKI. We hypothesized that treatment with terlipressin would, via the V1aR, protect renal function, modulate inflammation, and diminish NF-κB expression in renal tissue.

## Methods

### Ethical aspects

All experimental procedures were approved by the Medical and Research Ethics Committee of the University of São Paulo School of Medicine (Reference no. 008/16) and were conducted in accordance with the National Institutes of Health Guide for the Care and Use of Laboratory Animals. Male Wistar rats were obtained from the animal facilities of the University of São Paulo School of Medicine. The study is reported in accordance with the ARRIVE guidelines.

### Experimental protocol

Male Wistar rats were intubated and anesthetized with inhaled isoflurane, at 3% for induction and at 1% for maintenance throughout the experiment. The femoral artery was cannulated for blood withdrawal and for monitoring mean arterial pressure (MAP) during the experiment. The femoral vein was also cannulated for administration of the treatment and for the reinfusion of blood. The arterial and venous accesses were cleared with heparinized syringes.

In all of the rats in the experimental groups, HS was induced by pressure-controlled bleeding, targeting an MAP of 30–40 mmHg, and was maintained for 60 min. The quantity of blood withdrawn was limited to ≤ 60% of the total blood volume. Additional blood withdrawal or restitution of small volumes of blood was performed to maintain the target MAP throughout the protocol. After hemodynamic stabilization, the animals were separated into four groups: control (*n* = 6), in which the rats were not induced to HS and therefore received no fluid resuscitation or treatment; 3LR (*n* = 10), in which the rats received infusion of LR at 3 times the volume of the blood withdrawn; 2LR + TLP (*n* = 8), in which the rats received infusion of LR at 2 times the volume of the blood withdrawn, together with intravenous injection of terlipressin (Glypressin; Ferring Pharmaceuticals, Copenhagen, Denmark) at 10 µg/100 g body weight; and 1LR + TLP (*n* = 8), in which the rats received infusion of LR at a volume equal to that of the blood withdrawn, together with intravenous injection of terlipressin (as above). In the 3LR, 2LR + TLP, and 1LR + TLP groups, the fluid resuscitation, with or without terlipressin administration, was performed over a period of approximately 30 min, which was followed by a 15-min break with no intervention. All of the animals, except for the control group animals, then received all of the blood that had previously been withdrawn. The protocol ended after all of the blood had been given back to the animals. This entire treatment procedure was intended to mimic what happens in real life, when an injured patient is being rescued. The dosage of isoflurane was decreased gradually, and the animals were extubated. The animals were allowed to recover in heated beds for 4 h, after which they were moved to metabolic cages, where they remained for the collection of 24-h urine samples. The animals were then anesthetized with tribromoethanol 2.5% (2,2,2-tribromoethanol 97%; Sigma-Aldrich, Milwaukee, WI, USA), blood was collected, and the femoral artery was cannulated for perfusion of the kidneys with PBS. The kidneys were perfused with a peristaltic pump (Minipuls 3; Gilson, Middleton, WI, USA) at 8.4 mL/min. The kidneys were then removed.

### Analysis of blood and urine

Urine and blood samples were centrifuged in aliquots for 30 min at 4,000 g. Serum and urinary levels of sodium were measured with an EasyLyte Na/K Analyzer (Medica Corporation, Bedford, MA, USA). Creatinine and urea were measured with kits (Labtest Diagnóstica, Lagoa Santa, Brazil). Creatinine clearance was calculated by the following formula:

$$Creatinine\,clearance = \, \left[ {U_{creat} \times \, \left( {U_{volume} /T} \right)} \right]/P_{creat}$$

where U_creat_ is the urinary concentration of creatinine (in mg/dL), *U*_*volume*_ is the urine volume in microliters, *T* is the time in minutes, and *P*_*creat*_ is the plasma concentration of creatinine (in mg/dL).

### Urinary neutrophil gelatinase-associated lipocalin

Urinary neutrophil gelatinase-associated lipocalin (NGAL) was measured by using a commercially available ELISA kit (046; BioPorto Diagnostics, Gentofte, Denmark).

### Western Blot Analysis

#### Kidney Fractions

Kidney samples were homogenized in ice-cold isolation solution (200 mM mannitol, 80 mM HEPES, and 41 mM potassium hydroxide, pH 7.5) containing a protease inhibitor cocktail (Sigma Chemical, St Louis, MO, USA) in a homogenizer (PT 10/35; Brinkmann Instruments, Westbury, NY, USA), as previously described^31^. To remove nuclei and cell debris, homogenates were centrifuged at 4000×*g* for 30 min at 4 °C. Supernatants were isolated, and protein was quantified with a bicinchoninic acid protein assay kit (Pierce BCA Protein Assay Kit no. 23225; Thermo Fisher Scientific, Waltham, MA, USA).

#### Electrophoresis

Kidney samples were run on polyacrylamide minigels^29^. After transfer by electroelution to polyvinylidene difluoride membranes (GE Healthcare, Little Chalfont, UK), blots were blocked with 5% nonfat dry milk in Tris-buffered saline. Blots were then incubated overnight with antibodies against aquaporin 2 (AQP2, 1:500), V1aR (1:500), NF-κB (1:500), and β-actin (1:100 000), all of which were obtained from Santa Cruz Biotechnology (Dallas, TX, USA). The labeling was visualized with a horseradish peroxidase-conjugated secondary antibody (anti-mouse, 1:4,000; Sigma Chemical, St. Louis, MO, USA) and enhanced chemiluminescence detection (Amersham Pharmacia Biotech, Piscataway, New Jersey, USA).

We scanned the enhanced chemiluminescence films with an imaging system (Alliance 4.2; UVItec, Cambridge, UK). We then used densitometry to perform a quantitative analysis of the antibodies, normalizing the bands to β-actin expression^32^.

### Light microscopy

Four-micrometer histological sections of kidney tissue were stained with hematoxylin and eosin and examined under light microscopy. In 20 fields (0.087 mm^2^ each; magnification, × 400), we graded the proportional renal damage (tubular epithelial swelling, vacuolar degeneration, necrosis, and desquamation) as follows: 0 (< 5%); 1 (5–25%); 2 (26–50%); 3 (51–75%); or 4 (> 75%). To minimize bias in the morphometric analysis, the observer was blinded to the treatment groups. The mean scores were calculated by animal and by group.

### Immunohistochemistry

Histological sections of renal tissue were incubated for 1 h at room temperature with the following antibodies: anti-V1a (1:200; CUSABIO, College Park, MD, USA), anti-CD68 (1:200, Millipore, Billerica, MA, USA), anti-angiotensin (ANG) II type 1 (anti-AT-1, 1:50; Research Diagnostics Inc, Flanders, NJ, USA), anti-CD43 (1:50; Seralab, Crawley Down, UK), anti-Toll-like receptor 4 (anti-TLR4, 1:100; Santa Cruz Biotechnology, Dallas, TX, USA), and anti-uromodulin (1:100; Millipore, Billerica, MA, USA). The reaction product was detected with horseradish peroxidase-conjugated HRP System (Anti-rabbit Polymer; Dako, Glostrup, Denmark), and the color reaction was developed with 3,3-diaminobenzidine (Sigma-Aldrich, St. Louis, MO, USA). The histological sections were divided into 20 fields (0.087 mm^2^ each; magnification, × 400), and the mean value for each animal was presented and calculated with GraphPad Prism, version 5.03 (GraphPad Software Inc., San Diego, CA, USA).

### Cytokine analysis

To determine the levels of interleukin (IL)-18 and IL-6 in kidney tissue, we submitted samples to a multiplex cytokine assay (Bio-Plex Rat 9-Plex kit; Bio-Rad, Hercules, CA, USA). The assay was read on the Bio-Plex suspension array system (Bio-Rad), and the data were analyzed with GraphPad Prism, version 5.03 (GraphPad Software Inc., San Diego, CA, USA).

### Statistical analysis

Differences among the means of multiple parameters were analyzed by one-way analysis of variance, followed by the Student–Newman–Keuls test. Quantitative data are expressed as mean ± SEM, and values of *P* < 0.05 were considered statistically significant. Survival analyses were compared by a log-rank test. The statistical software used was GraphPad Prism, version 6.0.

## Results

### Blood withdrawal

There was no significant difference among the groups in terms of the quantity of blood withdrawn. On average, we withdrew 7.42 ± 0.61 mL of blood from the rats in the 3LR group, whereas we withdrew 6.93 ± 0.67 mL and 6.94 ± 0.70 mL from those in the 2LR + TLP and 1LR + TLP groups, respectively.

### Survival curve

**Figure 1:**
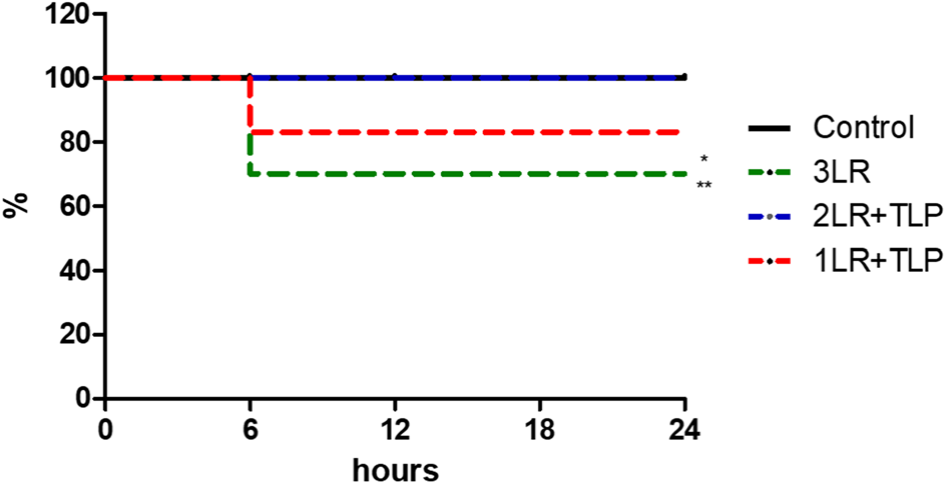
Curve of survival after hemorrhagic shock. *P < 0.05 vs. 2LR + TLP; **P < 0.01 vs. control; Control, no intervention; 3LR, induction of hemorrhagic shock, followed by infusion of lactated Ringer’s at 3 times the volume of the blood withdrawn; 2LR + TLP, induction of hemorrhagic shock, followed by infusion of lactated Ringer’s at 2 times the volume of the blood withdrawn, together with intravenous injection of terlipressin at 10 µg/100 g body weight; 1LR + TLP, induction of hemorrhagic shock, followed by infusion of lactated Ringer’s at a volume equal to that of the blood withdrawn, together with intravenous injection of terlipressin at 10 µg/100 g body weight.

Mortality was significantly higher in the 3LR group than in the 2LR + TLP and control groups. However, when we compared the 1LR + TLP group with the 3LR, 2LR + TLP, and control groups, we found no significant differences in mortality. As illustrated in Fig. 1, three (30.0%) of the 10 rats in the 3LR group died, compared with only one (12.5%) of the eight rats in the 1LR + TLP group and none of the eight rats in the 2LR + TLP group.

### MAP

**Figure 2:**
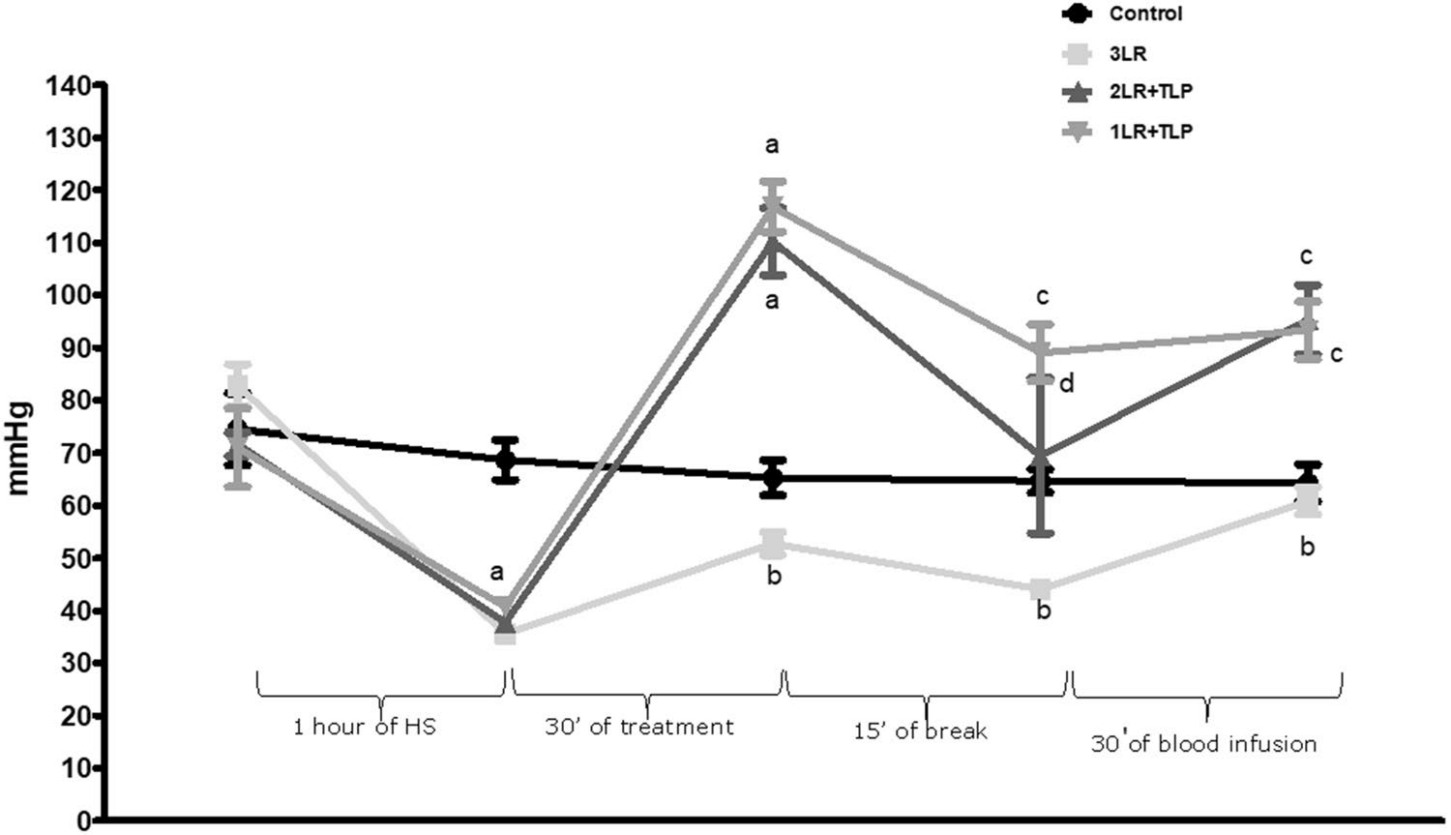
Mean arterial pressure during the experiment. ^a^*P* < 0.001 vs. control; ^b^*P* < 0.001 vs. 2LR + TLP and 1LR + TLP; ^c^*P* < 0.01 vs. control; ^d^*P* < 0.05 vs. control; HS, hemorrhagic shock; Control, no intervention; 3LR, induction of HS, followed by infusion of lactated Ringer’s at 3 times the volume of the blood withdrawn; 2LR + TLP, induction of HS, followed by infusion of lactated Ringer’s at 2 times the volume of the blood withdrawn, together with intravenous injection of terlipressin at 10 µg/100 g body weight; 1LR + TLP, induction of HS, followed by infusion of lactated Ringer’s at a volume equal to that of the blood withdrawn, together with intravenous injection of terlipressin at 10 µg/100 g body weight.

At baseline, there were no significant differences among the groups in terms of the MAP (Fig. 2). At the end of the HS-induction portion of the protocol, the mean MAP was significantly lower in the 3LR, 2LR + TLP, and 1LR + TLP groups than in the control group (37.6 ± 5.7 mmHg, 35.7 ± 1.9 mmHg, and 39.6 ± 2.4 mmHg, respectively, vs. 68.7 ± 9.3 mmHg; *P* < 0.001 for all). The MAP was measured again at the end of the 30-min period of treatment (with LR or LR plus terlipressin). At that point, the mean MAP was significantly higher in the 2LR + TLP and 1LR + TLP groups than in the 3LR and control groups (108 ± 6.4 and 114.1 ± 11.1 mmHg, respectively, vs. 59.3 ± 19.3 and 65.3 ± 8.3 mmHg, respectively; *P* < 0.001 for all). After the 15-min break and the reinfusion of the blood previously withdrawn, the mean MAP decreased in the 2LR + TLP and 1LR + TLP groups, although it was still higher in those two groups than in the 3LR and control groups (84.0 ± 12.7 and 88.4 ± 11.7 mmHg, respectively, vs. 50.7 ± 17.9 and 64.7 ± 5.4 mmHg, respectively; *P* < 0.05). At the end of the experimental protocol, the mean MAP was still higher in the 1LR + TLP and 2LR + TLP groups than in the 3LR and control groups (88.0 ± 15.6 and 91.0 ± 16.4 vs. 64.9 ± 10.9 and 64.0 ± 5.4 mmHg, respectively; *P* < 0.01 for all), as can also be seen in Fig. 2.

### Body weight

The 3LR group rats presented the most post-HS weight gain, which could be attributable to the fact that the amount of LR infused was greatest in that group. Nevertheless, there were no significant differences among the 3LR, 2LR + TLP, and 1LR + TLP groups in terms of the 24-h urine volume at 24 h after HS (Table 1).

**Table 1:** Characteristics of the animals at 24 h after the induction of hemorrhagic shock.

Variable	Group			
	Control	3LR	2LR + TLP	1LR + TLP
Urea (mg/dL)	41.2 ± 2.7	79.3 ± 15.6^a^	88.4 ± 10^a^	98.2 ± 5.4^b^
Creatinine (mg/dL)	0.58 ± 0.07	0.73 ± 0.06	0.67 ± 0.05	0.92 ± 0.09^a^
24-h urine volume (mL)	22.5 ± 5.6	26.4 ± 8.2	40.2 ± 6.4	38.4 ± 6.7
Creatinine clearance (mL/min)	1.22 ± 0.12	0.63 ± 0.17^b,c^	1.20 ± 0.13	0.75 ± 0.11
FeNa	0.20 ± 0.06	1.34 ± 0.41^a^	0.83 ± 0.20	0.69 ± 0.25
NGAL	0.48 ± 0.14	316.3 ± 152^d^	45.3 ± 14.5	55.1 ± 10.3
Body weight (g)	271 ± 26	375 ± 5.0^e^	275 ± 5.0	310 ± 15

Control, no intervention; 3LR, induction of hemorrhagic shock, followed by infusion of lactated Ringer’s at 3 times the volume of the blood withdrawn; 2LR + TLP, induction of hemorrhagic shock, followed by infusion of lactated Ringer’s at 2 times the volume of the blood withdrawn, together with intravenous injection of terlipressin at 10 µg/100 g body weight; 1LR + TLP, induction of hemorrhagic shock, followed by infusion of lactated Ringer’s at a volume equal to that of the blood withdrawn, together with intravenous injection of terlipressin at 10 µg/100 g body weight; FeNa, fractional excretion of sodium; NGAL, neutrophil gelatinase-associated lipocalin.

^a^*P* < 0.05 vs. control; ^b^*P* < 0.01 vs. control; ^c^*P* < 0.01 vs. 2LR + TLP; ^d^*P* < 0.01 vs. control, 2LR + TLP, and 1LR + TLP; ^e^*P* < 0.05 vs. control, 2LR + TLP, and 1LR + TLP.

### Renal function

**Figure 3:**
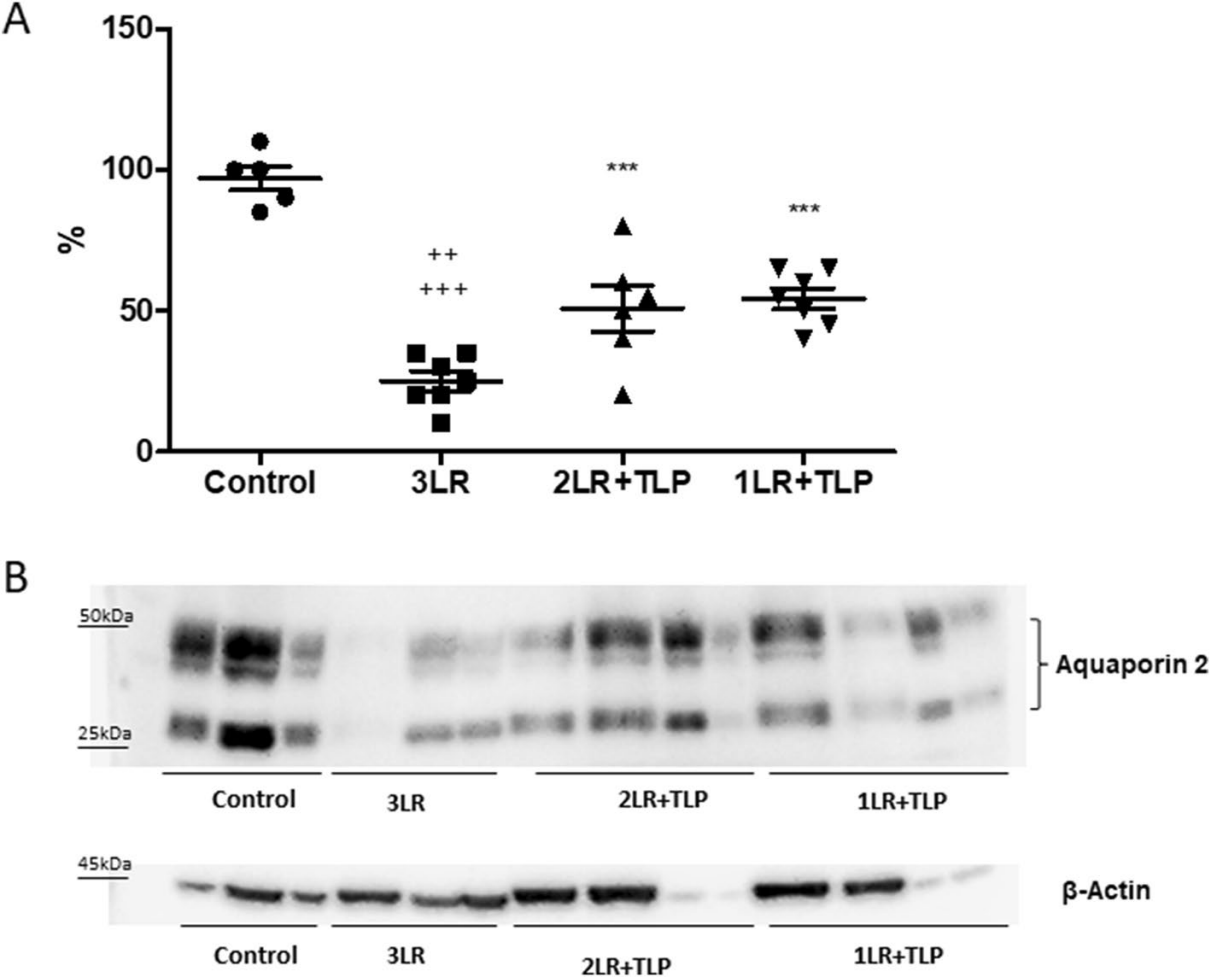
Aquaporin 2 expression in renal tissue at 24 h after hemorrhagic shock induction. Densitometric analysis (**A**) and immunoblotting (**B**). Immunoblots reacted with anti-AQP2 revealed 29- and 35 to 50-kD AQP2 bands, representing nonglycosylated and glycosylated forms of AQP2, respectively (Supplementary Fig. 1). ***P < 0.001 vs. control; ^++^*P* < 0.01 vs. 2LR + TLP; ^+++^*P* < 0.001 vs. 1LR + TLP; Control, no intervention; 3LR, induction of hemorrhagic shock, followed by infusion of lactated Ringer’s at 3 times the volume of the blood withdrawn; 2LR + TLP, induction of hemorrhagic shock, followed by infusion of lactated Ringer’s at 2 times the volume of the blood withdrawn, together with intravenous injection of terlipressin at 10 µg/100 g body weight; 1LR + TLP, induction of hemorrhagic shock, followed by infusion of lactated Ringer’s at a volume equal to that of the blood withdrawn, together with intravenous injection of terlipressin at 10 µg/100 g body weight.

**Figure 4:**
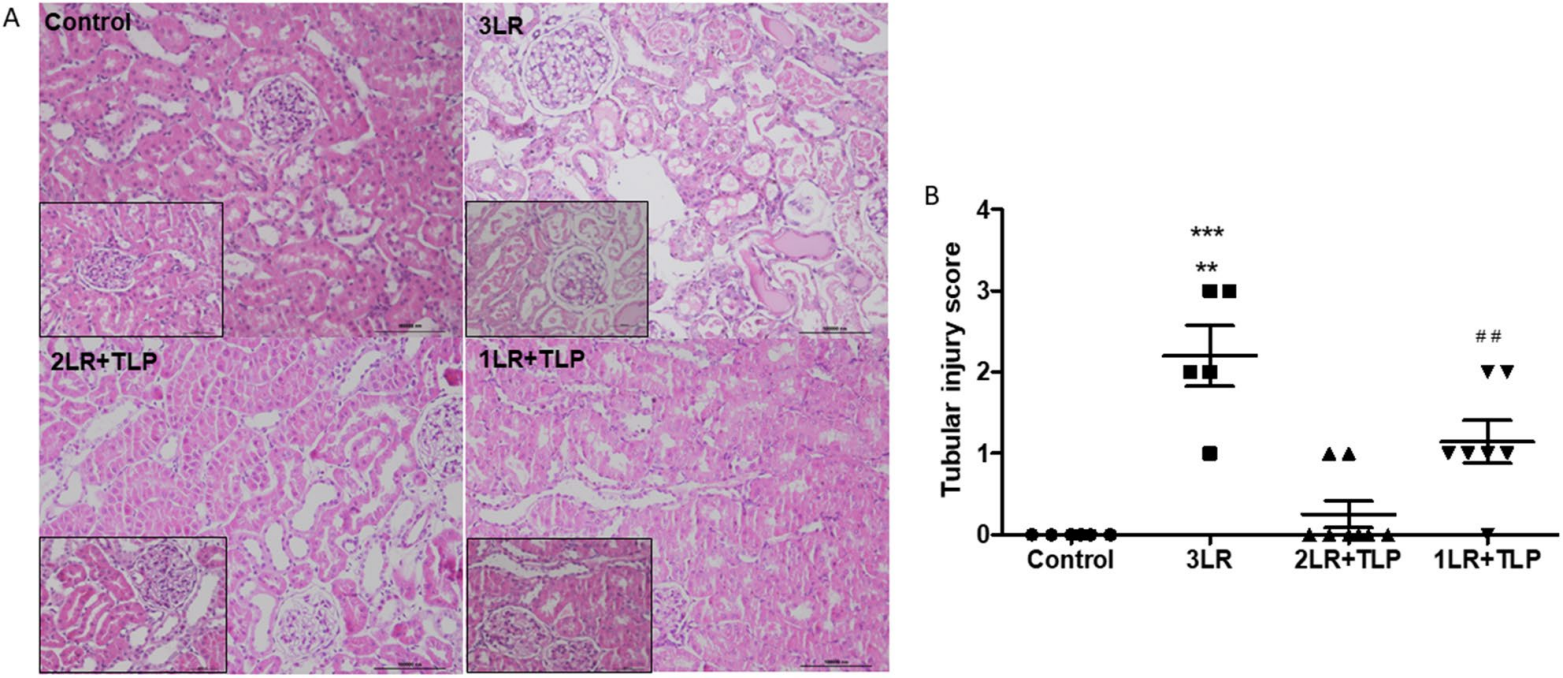
Acute tubular injury at 24 h after hemorrhagic shock induction. Kidney tissue sections stained with periodic acid-Schiff (A). Magnification, × 200 and × 400. Tubular damage score measured in the cortex (B). ***P < 0.001 vs. control and 2LR + TLP; **P < 0.01 vs. 1LR + TLP; ^##^*P* < 0.01 vs. control and 2LR + TLP; Control, no intervention; 3LR, induction of hemorrhagic shock, followed by infusion of lactated Ringer’s at 3 times the volume of the blood withdrawn; 2LR + TLP, induction of hemorrhagic shock, followed by infusion of lactated Ringer’s at 2 times the volume of the blood withdrawn, together with intravenous injection of terlipressin at 10 µg/100 g body weight; 1LR + TLP, induction of hemorrhagic shock, followed by infusion of lactated Ringer’s at a volume equal to that of the blood withdrawn, together with intravenous injection of terlipressin at 10 µg/100 g body weight.

Table 1 presents the renal function at 24 h after HS. The mean serum level of urea was lower in the control group than in all of the other groups. However, the glomerular filtration rate (as determined by measuring creatinine clearance) was preserved in the 2LR + TLP group and was comparable to that observed for the control group. No such protection was seen in the 1LR + TLP group. The mean creatinine clearance was lower in the 3LR group than in the control and 2LR + TLP groups. The mean fractional excretion of sodium and urinary NGAL expression were higher in the 3LR group than in the other groups. Semiquantitative immunoblotting (Fig. 3) revealed that renal expression of AQP2 was significantly lower in the 3LR group rats than in the control, 2LR + TLP, and 1LR + TLP group rats (25 ± 3.4% vs. 97 ± 4.4, 51 ± 8.2, and 54 ± 3.7%, respectively; *P* < 0.01 for all). However, as shown in Fig. 3, renal AQP2 expression was lower in the terlipressin-treated (2LR + TLP and 1LR + TLP) groups than in the control group (*P* < 0.001). Figure 4 shows that renal tubular damage (acute tubular necrosis) was more extensive in the 3LR group than in the control, 2LR + TLP, and 1LR + TLP groups (2.20 ± 0.40 vs. 0.00 ± 0.00, 0.25 ± 0.16, and 1.14 ± 0.30, respectively; *P* < 0.01 for all).

### TLR4/NF-κB signaling pathway

**Figure 5:**
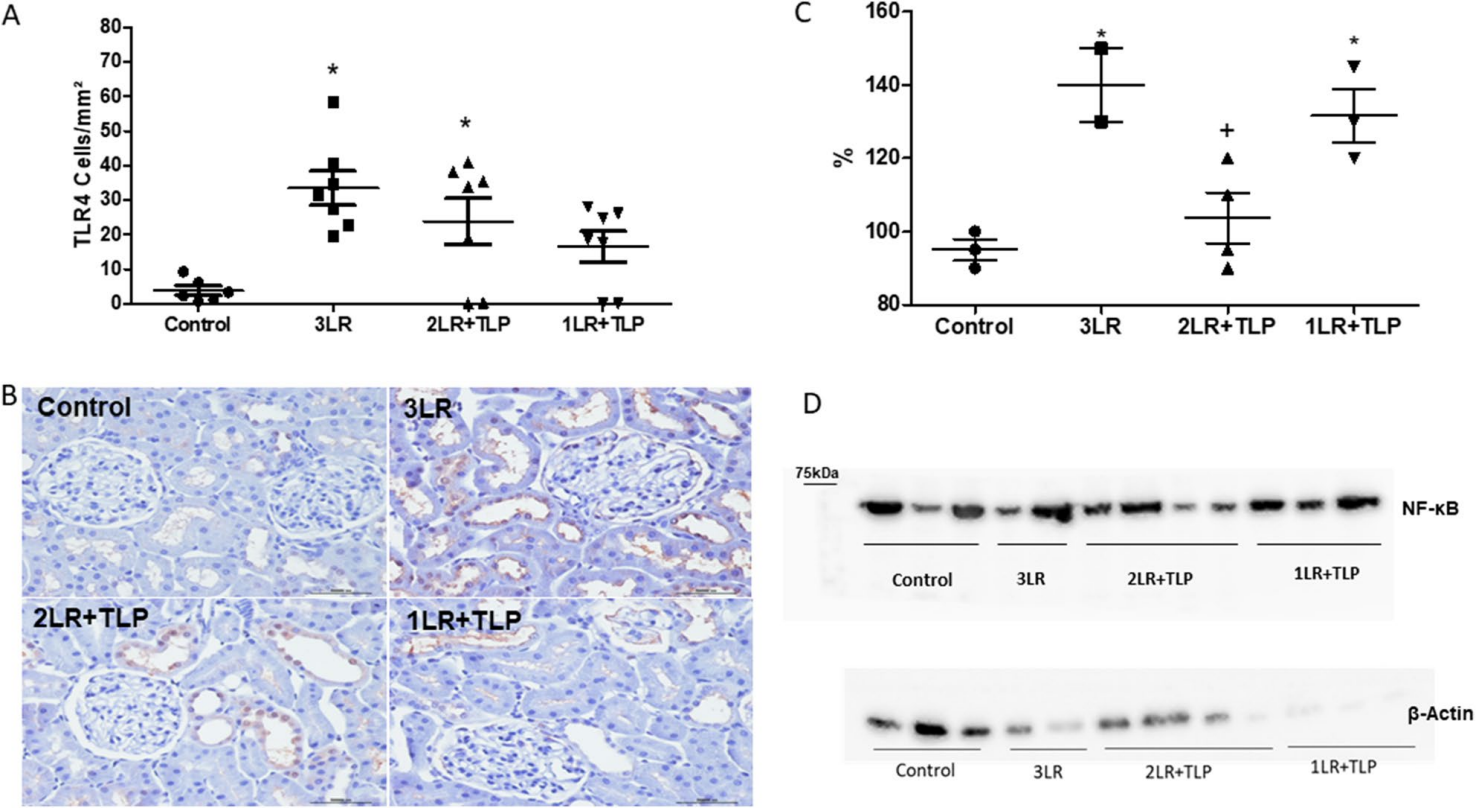
Immunohistochemical analysis of Toll-like receptor 4 (TLR4) expression in rat kidney tissue. (A) Bar graph of TLR4 expression. Immunostaining (brown, in B) for TLR4+ cells in kidney cortex samples from control, 3LR, 2LR + TLP, and 1LR + TLP group rats. Magnification, × 400. Densitometric analysis (C) and immunoblotting (D) of nuclear factor kappa B (NF-κB) expression in the renal tissue at 24 h after hemorrhagic shock induction (Supplementary Fig. 2). *P < 0.05 vs. control; ^+^*P* < 0.05 vs. 3LR and 1LR + TLP. Control, no intervention; 3LR, induction of hemorrhagic shock, followed by infusion of lactated Ringer’s at 3 times the volume of the blood withdrawn; 2LR + TLP, induction of hemorrhagic shock, followed by infusion of lactated Ringer’s at 2 times the volume of the blood withdrawn, together with intravenous injection of terlipressin at 10 µg/100 g body weight; 1LR + TLP, induction of hemorrhagic shock, followed by infusion of lactated Ringer’s at a volume equal to that of the blood withdrawn, together with intravenous injection of terlipressin at 10 µg/100 g body weight.

The increase in TLR4 expression in the renal interstitium, expressed as the mean number of TLR4+ cells/mm^2^, was significantly more pronounced in the 3LR and 2LR + TLP groups than in the control group (33.45 ± 4.95 and 23.92 ± 6.70 cells/mm^2^, respectively, vs. 3.82 ± 1.34 cells/mm^2^, *P* < 0.05), whereas the level of expression in the 1LR + TLP group (16.5 ± 4.50 cells/mm^2^) did not differ significantly from that seen in the other groups (Fig. 5A,B). Figure 5 C,D shows the densitometric analysis and immunoblotting of NF-κB expression in renal tissue at 24 h after HS induction. The mean NF-κB expression in renal tissue was higher in the 3LR and 1LR + TLP groups than in the control group (140 ± 10 and 132 ± 7.3%, respectively, vs. 95.0 ± 3.0%; *P* < 0.05), whereas it was lower in the 2LR + TLP group than in the 3LR and 1LR + TLP groups (104.0 ± 6.9% vs. 140 ± 10 and 132 ± 7.3%, respectively; *P* < 0.05).

### Cytokine levels

**Figure 6:**
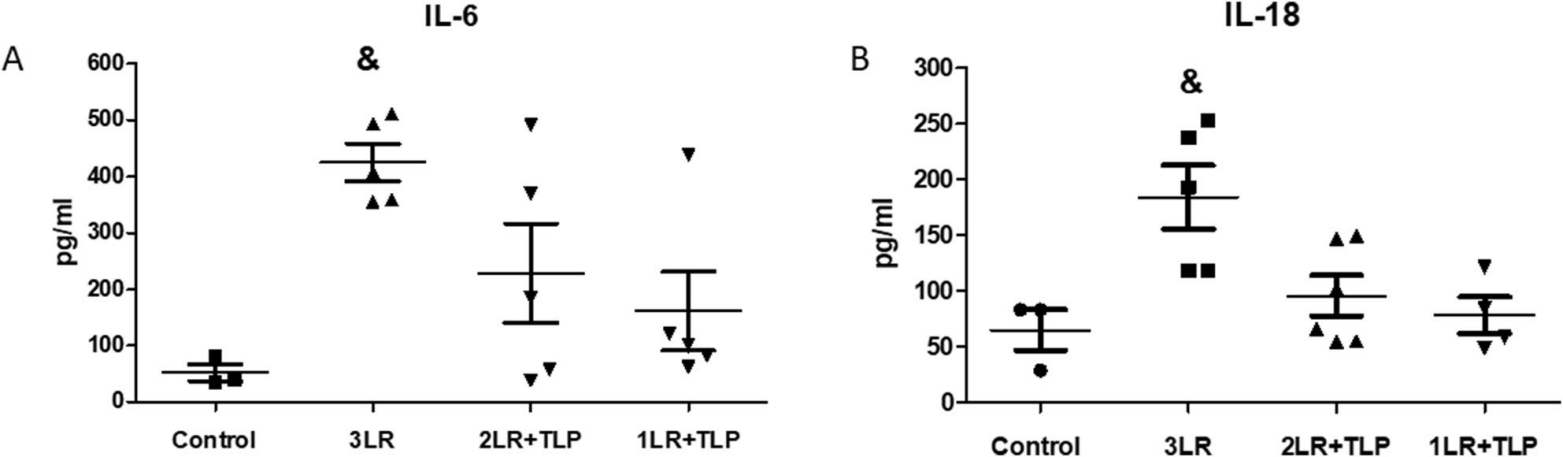
Cytokine levels in renal tissue at 24 h after hemorrhagic shock induction. Interleukin (IL)-6 (**A**) and IL-18 (**B**). ^&^*P* < 0.05 vs. the other groups. Control, no intervention; 3LR, induction of hemorrhagic shock, followed by infusion of lactated Ringer’s at 3 times the volume of the blood withdrawn; 2LR + TLP, induction of hemorrhagic shock, followed by infusion of lactated Ringer’s at 2 times the volume of the blood withdrawn, together with intravenous injection of terlipressin at 10 µg/100 g body weight; 1LR + TLP, induction of hemorrhagic shock, followed by infusion of lactated Ringer’s at a volume equal to that of the blood withdrawn, together with intravenous injection of terlipressin at 10 µg/100 g body weight.

As can be seen in Fig. 6, the levels of IL-6 and IL-18 in renal tissue were higher in the 3LR group than in the 2LR + TLP, 1LR + TLP, and control groups.

### Macrophage and CD43+ cell infiltration into renal tissue

**Figure 7:**
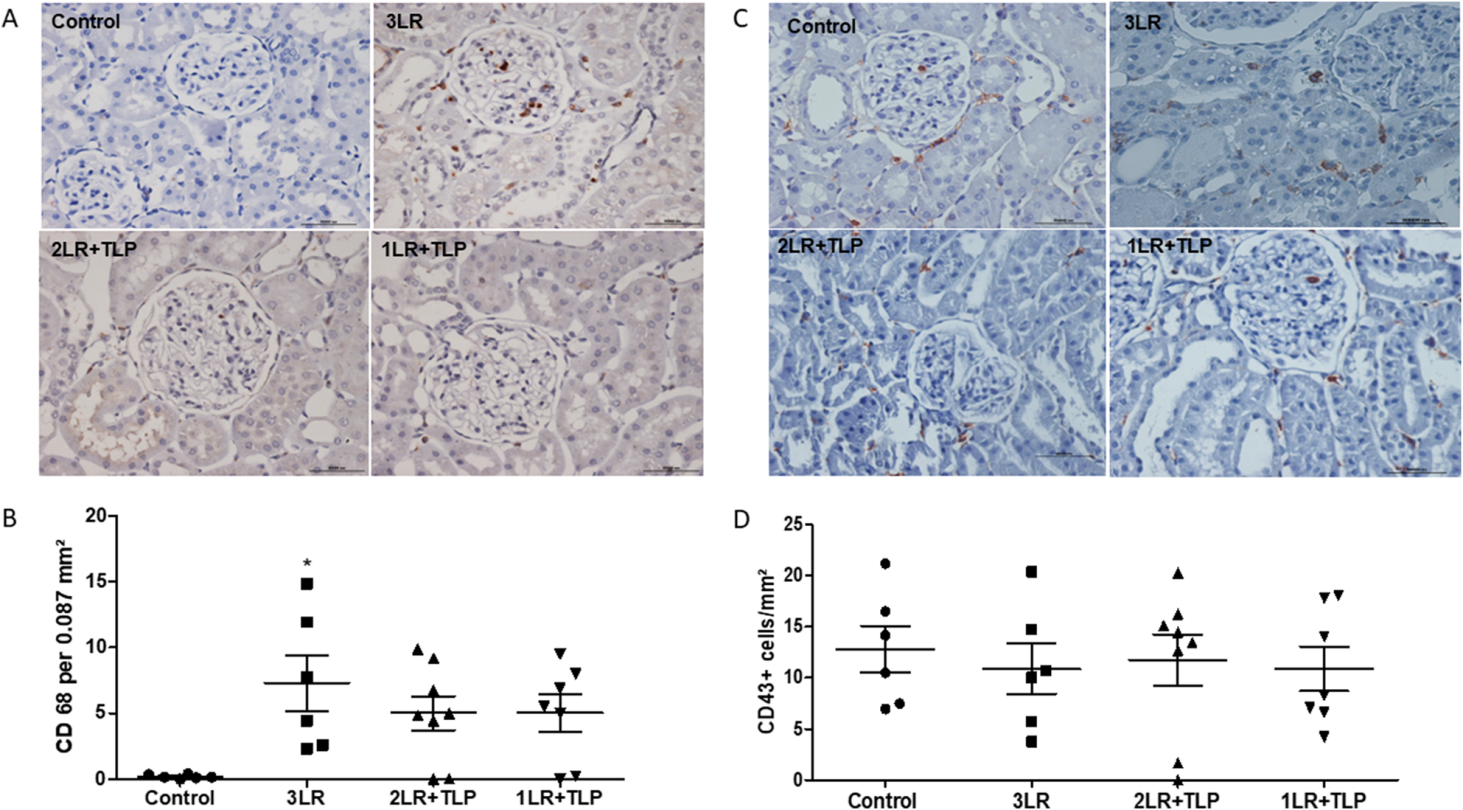
Immunohistochemical analysis of CD68 expression in rat kidney tissue. Immunostaining [brown, in (**A**)] for CD68 in kidney cortex samples from control, 3LR, 2LR + TLP, and 1LR + TLP group rats. Magnification, × 400. (**B**) Bar graph of CD68 expression. Immunohistochemical analysis of CD43 expression in rat kidney tissue. Immunostaining [brown, in (**C**)] for CD43 in kidney cortex samples from control, 3LR, 2LR + TLP, and 1LR + TLP group rats. Magnification, × 400. (**D**) Bar graph of CD43 expression. *P < 0.05 vs. control; Control, no intervention; 3LR, induction of hemorrhagic shock, followed by infusion of lactated Ringer’s at 3 times the volume of the blood withdrawn; 2LR + TLP, induction of hemorrhagic shock, followed by infusion of lactated Ringer’s at 2 times the volume of the blood withdrawn, together with intravenous injection of terlipressin at 10 µg/100 g body weight; 1LR + TLP, induction of hemorrhagic shock, followed by infusion of lactated Ringer’s at a volume equal to that of the blood withdrawn, together with intravenous injection of terlipressin at 10 µg/100 g body weight.

Macrophage infiltration into the renal interstitium, expressed as the mean number of CD68+ cells, was significantly higher in the 3LR group than in the control group (7.32 ± 2.11 vs. 0.20 ± 0.06 cells/mm^2^; *P* < 0.05). In addition, the 2LR + TLP and 1LR + TLP groups did not differ significantly from the control group in terms of the mean number of CD68+ cells in renal tissue (5.01 ± 1.3 and 5.02 ± 1.4 cells/mm^2^, respectively, vs. 0.20 ± 0.06 cells/mm^2^), as illustrated in Fig. 7A,B. However, there was no difference among the groups in terms of the infiltration of CD43+ cells into renal tissue (Fig. 7C,D).

### Uromodulin infiltration into renal tissue

**Figure 8:**
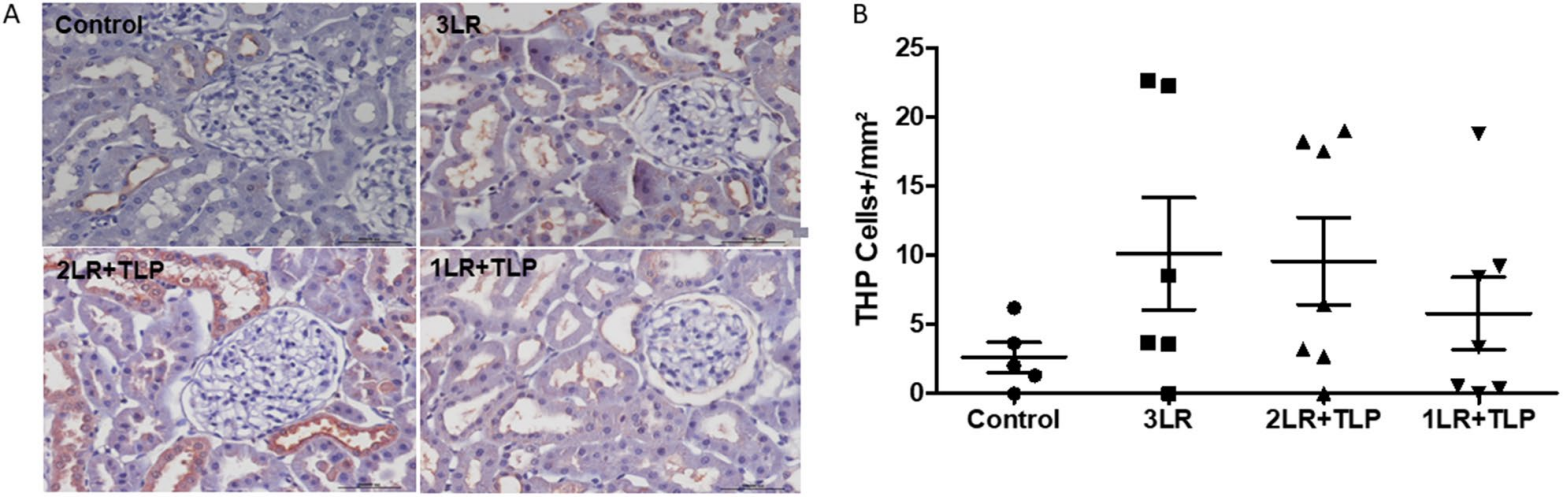
Immunohistochemical analysis of uromodulin expression in rat kidney tissue. Immunostaining [brown, in (**A**)] for uromodulin+ cells in kidney cortex samples from control, 3LR, 2LR + TLP, and 1LR + TLP group rats. Magnification, × 400. (**B**) Bar graph of uromodulin expression. Control, no intervention; 3LR, induction of hemorrhagic shock, followed by infusion of lactated Ringer’s at 3 times the volume of the blood withdrawn; 2LR + TLP, induction of hemorrhagic shock, followed by infusion of lactated Ringer’s at 2 times the volume of the blood withdrawn, together with intravenous injection of terlipressin at 10 µg/100 g body weight; 1LR + TLP, induction of hemorrhagic shock, followed by infusion of lactated Ringer’s at a volume equal to that of the blood withdrawn, together with intravenous injection of terlipressin at 10 µg/100 g body weight.

The increase in the expression of uromodulin (also known as Tamm–Horsfall protein) in the renal interstitium, expressed as the mean number of uromodulin+ cells, was more pronounced in the 3LR, 2LR + TLP, and 1LR + TLP groups than in the control group (10.13 ± 4.05, 9.60 ± 3.14, and 5.83 ± 2.60 cells/mm^2^, respectively, vs. 2.64 ± 1.70 cells/mm^2^), although the difference was not significant (Fig. 8).

### V1aR and AT-1 Receptor Expression in Renal Tissue

**Figure 9:**
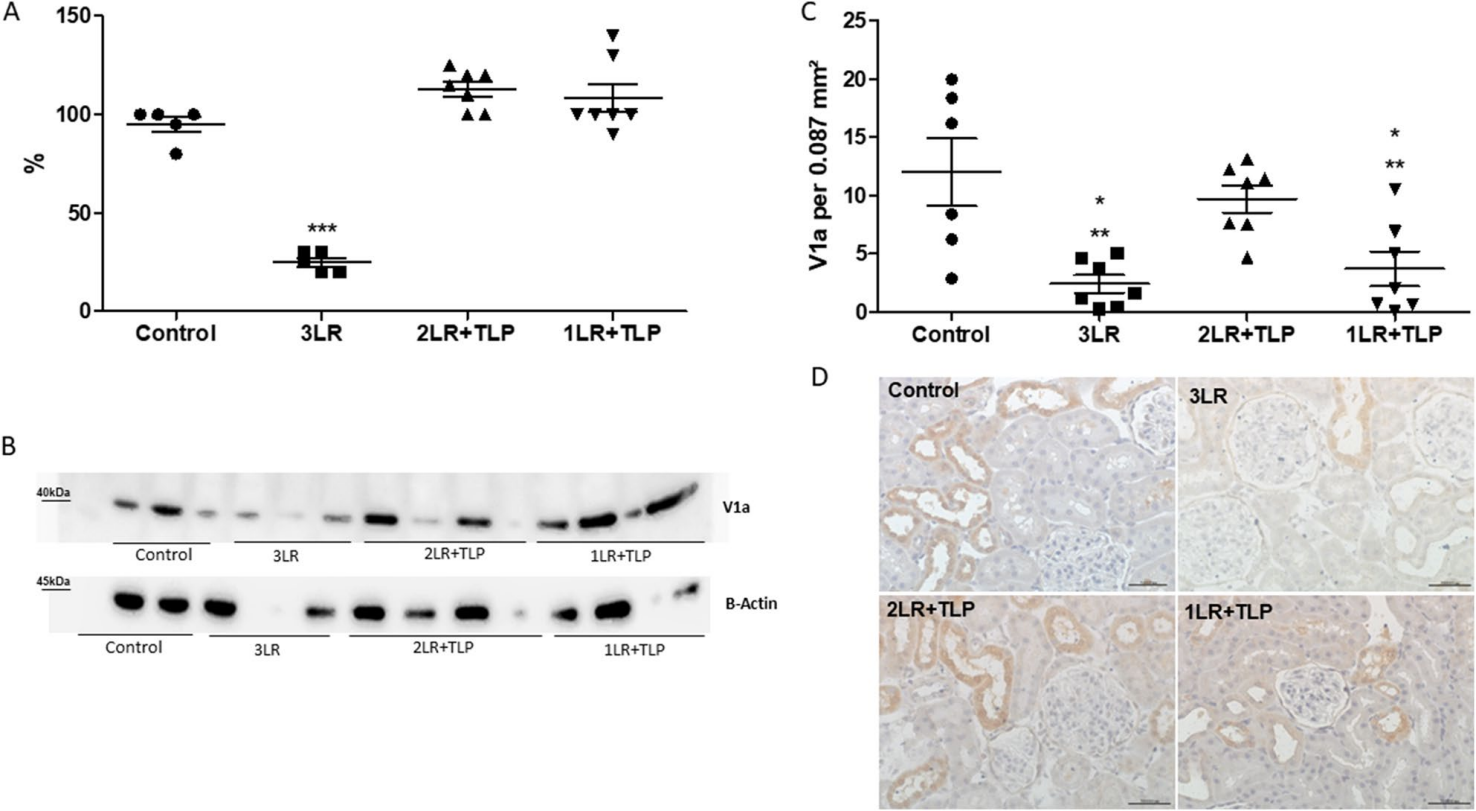
Arginine vasopressin 1a receptor (V1aR) expression in the renal tissue at 24 h after hemorrhagic shock induction. Immunohistochemical analysis of V1aR expression in rat kidney tissue at 24 h after hemorrhagic shock induction, by densitometric analysis (**A**) and immunoblotting (**B**) (Supplementary Fig. 3). Bar graph of V1aR expression (**C**) and immunostaining [brown, in (**D**)] for V1aR in kidney cortex samples from control, 3LR, 2LR + TLP, and 1LR + TLP group rats. Magnification, × 40. ***P < 0.001 vs. control, 2LR + TLP, and 1LR + TLP. *P < 0.05 vs. 2LR + TLP; **P < 0.01 vs. control; Control, no intervention; 3LR, induction of hemorrhagic shock, followed by infusion of lactated Ringer’s at 3 times the volume of the blood withdrawn; 2LR + TLP, induction of hemorrhagic shock, followed by infusion of lactated Ringer’s at 2 times the volume of the blood withdrawn, together with intravenous injection of terlipressin at 10 µg/100 g body weight; 1LR + TLP, induction of hemorrhagic shock, followed by infusion of lactated Ringer’s at a volume equal to that of the blood withdrawn, together with intravenous injection of terlipressin at 10 µg/100 g body weight.

As can be seen in Fig. 9, renal V1aR expression was markedly lower in 3LR group rats than in control, 2LR + TLP, and 1LR + TLP group rats (25.0 ± 2.2% vs. 95 ± 3.9, 113 ± 3.7, and 108 ± 7.0%, respectively, *P* < 0.001), although there was no significant difference among the control, 2LR + TLP, and 1LR + TLP group rats. However, the number of cells staining for V1aR was significantly lower in 3LR and 1LR + TLP group rats than in control and 2LR + TLP group rats (2.4 ± 0.7 and 3.7 ± 1.5 cells/0.087 mm^2^, respectively, vs. 12.0 ± 2.9 and 9.7 ± 1.2 cells/0.087 mm^2^, respectively; *P* < 0.05). There was no significant difference between the control and 2LR + TLP groups.

**Figure 10:**
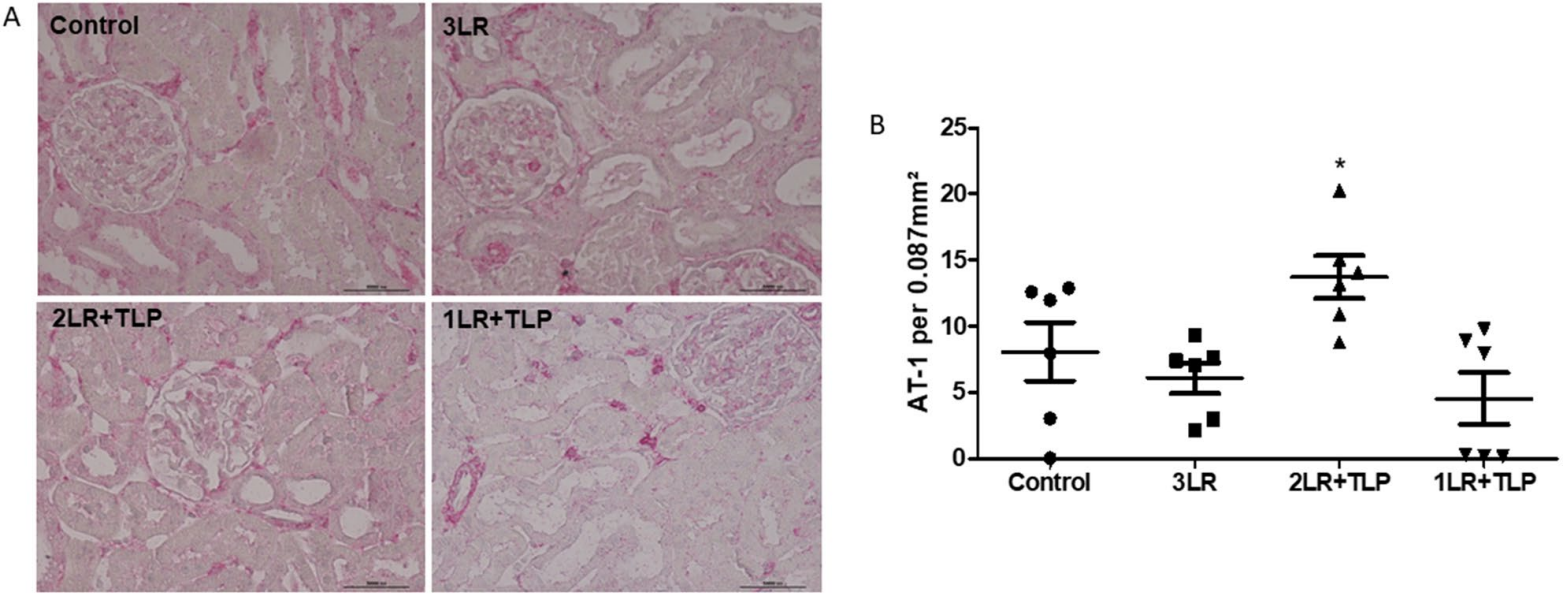
Immunohistochemical analysis of angiotensin II type 1 receptor (AT-1R) expression in rat kidney tissue. Immunostaining [violet, in (**A**)] for AT-1R in kidney cortex samples from control, 3LR, 2LR + TLP, and 1LR + TLP group rats. Magnification, × 400. (**B**) Bar graph of AT-1R expression. *P < 0.05 vs. control, 3LR and 1LR + TLP; Control, no intervention; 3LR, induction of hemorrhagic shock, followed by infusion of lactated Ringer’s at 3 times the volume of the blood withdrawn; 2LR + TLP, induction of hemorrhagic shock, followed by infusion of lactated Ringer’s at 2 times the volume of the blood withdrawn, together with intravenous injection of terlipressin at 10 µg/100 g body weight; 1LR + TLP, induction of hemorrhagic shock, followed by infusion of lactated Ringer’s at a volume equal to that of the blood withdrawn, together with intravenous injection of terlipressin at 10 µg/100 g body weight.

As shown in Fig. 10, the number of cells staining for the AT-1 receptor (AT-1R) was significantly higher in the 2LR + TLP group than in the control, 3LR, and 1LR + TLP groups (13.7 ± 1.6 cells/0.087 mm^2^ vs. 8.1 ± 2.2, 6.1 ± 1.2 and 4.5 ± 1.9 cells/0.087 mm^2^, respectively; *P* < 0.05). The number of cells staining for AT-1R was similar in the control, 3LR, and 1LR + TLP groups.

## Discussion

Here, we have established that 60 min of pressure-controlled HS induces AKI and histologic lesions of acute tubular necrosis in rats. Generating standardized animal models to mimic a clinically relevant situation of HS has proven to be difficult^33,34^. That was even more difficult in our study because we evaluated the animals at 24 h after HS induction. In addition, we have demonstrated that the therapeutic use of terlipressin accompanied by conservative LR resuscitation reduced mortality and protected against the inflammatory response in the kidneys. That protection might be regulated by decreased expression of the NF-κB pathway and consequent attenuation of the inflammatory process. We find it interesting that V1aR expression was normalized in the 2LR + TLP group at 24 h after terlipressin administration.

Trauma-induced HS is one of the leading causes of death among young people in large cities in low- and middle-income countries^35,36^. During the pre-hospitalization period, hemorrhage is a contributing factor in 33–56% of all trauma-related deaths and is the leading cause of death among trauma victims found dead upon the arrival of emergency medical services^37^. In the metropolitan area of São Paulo, in Brazil, trauma-induced HS is a major cause of AKI and death among young people^38^. Currently, aggressive LR administration is widely used in the resuscitation of patients with hypovolemic shock, including HS^39,40^. Standard resuscitation practice for HS mandates the use of high volumes of crystalloids. However, that practice can have adverse effects, such as interstitial edema in various organs^41^, an increase in the pro-inflammatory cytokine profile^5^, and increased intracranial pressure^42^. Lee et al. demonstrated that vasopressor use as an adjunct therapy is associated with less lung edema and a favorable inflammatory cytokine profile. In the present study, rats in the 3LR group presented higher body weights in comparison with those in the other groups, although 24-h urine volume after HS induction was comparable among the groups. The increased body weight might be related to an increase in whole-body edema. We found that terlipressin administration and LR infusion that was more conservative was able to prevent the edema caused by high-volume fluid resuscitation. Previous studies conducted by our group in pigs demonstrated that during recovery from HS-induced hypotension, terlipressin was effective in normalizing cerebral perfusion pressure, decreasing edema, and normalizing cerebral markers of water balance^6^. In an ovine model of sepsis, Maybauer et al. demonstrated that selepressin (a V1aR agonist) decreased the cumulative fluid intake, with a cumulative fluid balance near zero^43^. The reduction in fluid accumulation resulting from selepressin administration was accompanied by a significant blunting of the sepsis-induced drops in plasma total protein concentration and oncotic pressure. The authors concluded that resuscitation with the selective V1aR agonist blocked vascular leakage. Selepressin decreased fluid overload due to pulmonary capillary leak, a major prognostic factor for mortality among intensive care unit patients with sepsis^44^.

Terlipressin, a V1aR agonist, is inactive in its native form but is transformed into the biologically active form, lysine-vasopressin, through enzymatic cleavage of glycyl residues by tissue peptidases^45^. Because terlipressin can be given in a single bolus, it is suitable for use in patients who live far from the trauma center. Terlipressin has been studied as a vasoactive drug for the management of catecholamine-resistant arterial hypotension in septic shock^46^, liver failure^25^, and acute gastrointestinal bleeding^26^. The effects of terlipressin consist of vasoconstrictor activity in vascular smooth muscle cells and pronounced vasoconstriction within the splanchnic circulation, having been shown to redistribute blood flow in order to restore perfusion pressure to organs such as the liver, kidneys, and brain^27^, as well as to increase survival rates in animal models of HS^47^. In the HS model employed in the present study, we found that treatment with terlipressin and conservative fluid management increased post-HS survival. Whether the mechanisms of the increase in survival after HS induction are related to the increase in MAP or to protection of the endothelium against vascular leakage has yet to be elucidated. Because we did not test different doses of terlipressin; we cannot determine whether that response is dose-dependent.

We found that treatment with terlipressin and conservative fluid management protected renal function, as measured by creatinine clearance, urinary NGAL, and expression of the water protein transporter in the renal tubules. In a previous study, our group demonstrated that terlipressin protected renal function at 120-min after HS induction in pigs^29^. In the present study, we have demonstrated that terlipressin has longer-lasting beneficial effects on renal function. It is noteworthy that although treatment with terlipressin was able to protect the renal function and histological damage in kidney tissue, that protection was much more pronounced in the 2LR + TLP group than in 1LR + TLP group, as evidenced by the tubular necrosis scores and the creatinine clearance levels. We speculate that fluid resuscitation is still mandatory in HS.

Because renal ischemia/reperfusion injury triggers an inflammatory cascade within the renal parenchyma, suppression of inflammatory responses might be a therapeutic approach that would protect renal tissue^9,48^. Ischemia/reperfusion injury induces renal production of pro-inflammatory cytokines such as IL-18^49^, and inflammatory mediators lead to activation of the TLR4/NF-κB signaling pathway, which plays a key role in inflammation and immunity. In the present study, terlipressin treatment restored renal expression of NF-κB, IL-18, IL-6 as well as reducing CD68 infiltration^16^. However, there was no difference among the groups in terms of CD43 infiltration. Lymphocyte infiltration began as early as 1 h after ischemia/reperfusion and appeared to peak at approximately 5 days thereafter^50^. Therefore, we might have observed some difference among the groups if they had been studied at 4 to 5 days after HS induction. It has previously been demonstrated that TLR4 mediates renal ischemia/reperfusion injury^51^. Activation of the TLR4 signaling pathway initiates activation of NF-κB^52^. In the present study, we were unable to demonstrate any significant difference between the terlipressin-treated animals and those in the 3LR group in terms of TLR4 expression, although the downstream signaling pathways (NF-κB and interleukins) differed among the groups. Uromodulin is a glycoprotein expressed exclusively by renal tubular cells lining the thick ascending limb of the loop of Henle^53^. It is frequently used as a marker of cortical and medullary thick ascending limb renal segments, all of which strongly express TLR4. Expression of uromodulin has also been shown to be significantly higher in those segments in an animal model of renal ischemia/reperfusion^54^. In the present study, we demonstrated an increase in uromodulin expression in HS, which is a novel finding. Zhao et al. investigated the functional significance of the complex signaling cascade activated by a V1 agonist ([Phe^2^, Orn^8^]-oxytocin) in astrocytes by assessing the impact of that agonist on the immune function of the astrocytes, focusing on regulation of pro-inflammatory cytokine production. The results of their analyses indicate that the V1 agonist studied suppresses the expression of IL-1B and tumor necrosis factor alpha at the mRNA and secreted peptide levels^30^. The authors suggested that the V1 agonist acts as an immunomodulator to repress pro-inflammatory cytokine expression in astrocytes and that a V1 agonist could exert an anti-inflammatory effect in vivo. Whether V1aR activation by terlipressin has an anti-inflammatory effect in HS-induced AKI still needs to be elucidated. The role of the renin–angiotensin–aldosterone system in maintaining blood pressure during the HS is well established^55^. In the present study, we found that AT-1R expression at 24 h after HS induction was highest in the 2LR + TLP group. That may reflect a regulatory response to intrarenal ANG II concentrations. Aoyagi et al. found that plasma renin activity and ANG II levels were lower in V1aR-knockout mice than in wild-type mice under basal and water-restricted conditions, implying that renin–angiotensin activity was suppressed in the knockout mice^56^. It has been known that V1aR activation stimulates renin–angiotensin activity and aldosterone release^57^. In our study, terlipressin normalized V1aR expression, as well as increasing AT-1R expression, by 24 h after HS.

One drawback of our study is that the mean arterial pressure was higher among the terlipressin-treated animals than among those in the control and 3LR groups. In cases of trauma, such hypertension could be deleterious because it can augment bleeding.

In conclusion, terlipressin combined with conservative fluid management could be a viable therapy for HS-induced AKI. Terlipressin might attenuate AKI by modulating the inflammatory response via the V1aR.

## Supplementary Information

Supplementary Figure S1.

Supplementary Figure S2.

Supplementary Figure S3.

## References

[1] Jacob M, Kumar P (2014). The challenge in management of hemorrhagic shock in trauma. Med. J. Armed Forces India..

[2] Nandra KK (2013). Pharmacological preconditioning with erythropoietin attenuates the organ injury and dysfunction induced in a rat model of hemorrhagic shock. Dis. Model Mech..

[3] Gutierrez G, Reines HD, Wulf-Gutierrez ME (2004). Clinical review: hemorrhagic shock. Crit. Care..

[4] Hostmann A (2008). Biphasic onset of splenic apoptosis following hemorrhagic shock: Critical implications for Bax, Bcl-2, and Mcl-1 proteins. Crit. Care..

[5] Lee CC (2014). A comparison of vasopressin, terlipressin and lactated ringers for resuscitation of uncontrolled hemorrhagic shock in an animal model. PLoS ONE.

[6] Ida KK (2015). Effects of terlipressin as early treatment for protection of brain in a model of haemorrhagic shock. Crit. Care..

[7] Powell RD (2005). MitoQ modulates oxidative stress and decreases inflammation following hemorrhage. J. Trauma Acute Care Surg..

[8] Cannon JW (2018). Hemorrhagic shock. N. Engl. J. Med..

[9] Jang HR, Ko GJ, Wasowska BA, Rabb H (2009). The interaction between ischemia-reperfusion and immune responses in the kidney. J. Mol. Med (Berl).

[10] Sharfuddin AA, Molitoris BA (2011). Pathophysiology of ischemic acute kidney injury. Nat. Rev. Nephrol..

[11] Mayeur N (2011). Morphologic and functional renal impact of acute kidney injury after prolonged hemorrhagic shock in mice. Crit. Care Med..

[12] Markó L (2016). Tubular epithelial NF-kappaB activity regulates ischemic AKI. J. Am. Soc. Nephrol..

[13] Moore-Olufemi SD (2005). Resuscitation-induced gut edema and intestinal dysfunction. J. Trauma..

[14] Kohama K (2015). Hydrogen inhalation protects against acute lung injury induced by hemorrhagic shock and resuscitation. Surgery.

[15] Gong H (2003). Reduced renal expression of AQP2, p-AQP2 and AQP3 in haemorrhagic shock-induced acute renal failure. Nephrol. Dial. Transplant..

[16] Zhao XG (2015). Ideal target arterial pressure after control of bleeding in a rabbit model of severe traumatic hemorrhagic shock: results from volume loading-based fluid resuscitation. J. Surg. Res..

[17] Bahrami S (2006). Small-volume fluid resuscitation with hypertonic saline prevents inflammation but not mortality in a rat model of hemorrhagic shock. Shock.

[18] Jin G (2012). Traumatic brain injury and hemorrhagic shock: evaluation of different resuscitation strategies in a large animal model of combined insults. Shock.

[19] Cantle PM, Cotton BA (2017). Balanced resuscitation in trauma management. Surg. Clin. North Am..

[20] Silva J, Gonçalves L, Sousa PP (2018). Fluid therapy and shock: An integrative literature review. Br. J. Nurs..

[21] Girard TD, Bernard GR (2007). Mechanical ventilation in ARDS: A state-of-the-art review. Chest.

[22] Wiedemann HP (2006). Comparison of two fluid-management strategies in acute lung injury. N. Engl. J. Med..

[23] Terada Y, Tomita K, Nonoguchi H, Yang T, Marumo F (1993). Different localization and regulation of two types of vasopressin receptor messenger RNA in microdissected rat nephron segments using reverse transcription polymerase chain reaction. J. Clin. Invest..

[24] Aoyagi T (2008). Vasopressin regulates the renin-angiotensin-aldosterone system via V1a receptors in macula densa cells. Am. J. Physiol. Renal Physiol..

[25] Eefsen M (2007). Comparison of terlipressin and noradrenalin on cerebral perfusion, intracranial pressure and cerebral extracellular concentrations of lactate and pyruvate in patients with acute liver failure in need of inotropic support. J. Hepatol..

[26] Levacher S (1995). Early administration of terlipressin plus glyceryl trinitrate to control active upper gastrointestinal bleeding in cirrhotic patients. Lancet.

[27] Narahara Y (2009). Effects of terlipressin on systemic, hepatic and renal hemodynamics in patients with cirrhosis. J. Gastroenterol. Hepatol..

[28] Stadlbauer KH (2003). Survival with full neurologic recovery after prolonged cardiopulmonary resuscitation with a combination of vasopressin and epinephrine in pigs. Anesth. Analg..

[29] Cardoso de Castro LU (2016). Vasopressin analog terlipressin attenuates kidney injury in hemorrhagic shock. Trauma Surg. Acute Care Open..

[30] Zhao L, Brinton RD (2004). Suppression of proinflammatory cytokines interleukin-1beta and tumor necrosis factor-alpha in astrocytes by a V1 vasopressin receptor agonist: A cAMP response element-binding protein-dependent mechanism. J. Neurosci..

[31] Cóndor JM (2016). Treatment with human wharton's jelly-derived mesenchymal stem cells attenuates sepsis-induced kidney injury, liver injury, and endothelial dysfunction. Stem Cells Transl. Med..

[32] Moreira RS (2020). Synthetic apolipoprotein A-I mimetic peptide 4F protects hearts and kidneys after myocardial infarction. Am. J. Physiol. Regul. Integr. Comp. Physiol..

[33] Tsukamoto T, Pape HC (2009). Animal models for trauma research: what are the options?. Shock.

[34] Singh AP (2012). Animal models of acute renal failure. Pharmacol. Rep..

[35] Bommakanti K, Feldhaus I, Motwani G, Dicker RA, Juillard C (2018). Trauma registry implementation in low- and middle-income countries: challenges and opportunities. J. Surg. Res..

[36] Laytin AD (2017). Comparing traditional and novel injury scoring systems in a US level-I trauma center: an opportunity for improved injury surveillance in low- and middle-income countries. J. Surg. Res..

[37] Sauaia A (1995). Epidemiology of trauma deaths: A reassessment. J. Trauma..

[38] Ministério da Saúde. Secretaria de Vigilância em Saúde. Sistema de Informação sobre Mortalidade. http://tabnet.datasus.gov.br (2021).

[39] Rohrig R (2012). Adverse effects of resuscitation with lactated ringer compared with ringer solution after severe hemorrhagic shock in rats. Shock.

[40] Phillips CR (2009). Resuscitation of haemorrhagic shock with normal saline vs. lactated Ringer's: Effects on oxygenation, extravascular lung water and haemodynamics. Crit. Care..

[41] Moon PF, Hollyfield-Gilbert MA, Myers TL, Kramer GC (1994). Effects of isotonic crystalloid resuscitation on fluid compartments in hemorrhaged rats. Shock.

[42] Cavus E (2009). Cerebral effects of three resuscitation protocols in uncontrolled haemorrhagic shock: A randomised controlled experimental study. Resuscitation.

[43] Maybauer MO (2014). The selective vasopressin type 1a receptor agonist selepressin (FE 202158) blocks vascular leak in ovine severe sepsis. Crit. Care Med..

[44] Vincent JL (2006). Sepsis in European intensive care units: results of the SOAP study. Crit. Care Med..

[45] Narahara Y (2009). Effects of terlipressin on systemic, hepatic and renal hemodynamics in patients with cirrhosis. J. Gastroenterol. Hepatol..

[46] Morelli A (2009). Continuous terlipressin versus vasopressin infusion in septic shock (TERLIVAP): A randomized, controlled pilot study. Crit. Care..

[47] Gil-Anton J (2020). Addition of terlipressin to initial volume resuscitation in a pediatric model of hemorrhagic shock improves hemodynamics and cerebral perfusion. PLoS ONE.

[48] Stroo I (2010). Chemokine expression in renal ischemia/reperfusion injury is most profound during the reparative phase. Int. Immunol..

[49] Bonventre JV, Yang L (2011). Cellular pathophysiology of ischemic acute kidney injury. J. Clin. Invest..

[50] Jang HR, Rabb H (2015). Immune cells in experimental acute kidney injury. Nat. Rev. Nephrol..

[51] Pulskens WP (2008). Toll-like receptor-4 coordinates the innate immune response of the kidney to renal ischemia/reperfusion injury. PLoS ONE.

[52] Hu X (2020). Inhibition of myeloid differentiation protein 2 attenuates renal ischemia/reperfusion-induced oxidative stress and inflammation via suppressing TLR4/TRAF6/NF-kB pathway. Life Sci..

[53] Schaeffer C, Devuyst O, Rampoldi L (2021). Uromodulin: Roles in Health and Disease. Annu. Rev. Physiol..

[54] Micanovic R (2018). Tamm-Horsfall protein regulates mononuclear phagocytes in the kidney. J. Am. Soc. Nephrol..

[55] Milanez MIO, Martins GR, Nishi EE, Bergamaschi CT, Campos RR (2020). Differential sympathetic vasomotor control by spinal AT 1 and V1a receptors in the acute phase of hemorrhagic shock. Eur. J. Pharmacol..

[56] Aoyagi T, Koshimizu T-A, Tanoue A (2009). Vasopressin regulation of blood pressure and volume: findings from V1a receptor-deficient mice. Kidney Int..

[57] Szczepanska-Sadowska E, Czarzasta K, Cudnoch-Jedrzejewska A (2018). Dysregulation of the renin-angiotensin system and the vasopressinergic system interactions in cardiovascular disorders. Curr. Hypertens. Rep..

