# Supplementary figures and images for: Terlipressin combined with conservative fluid management attenuates hemorrhagic shock-induced acute kidney injury in rats
Source: Sci Rep. 2022 Nov 28;12:20443. doi: 10.1038/s41598-022-24982-0 (PMC9705717; doi:10.1038/s41598-022-24982-0)

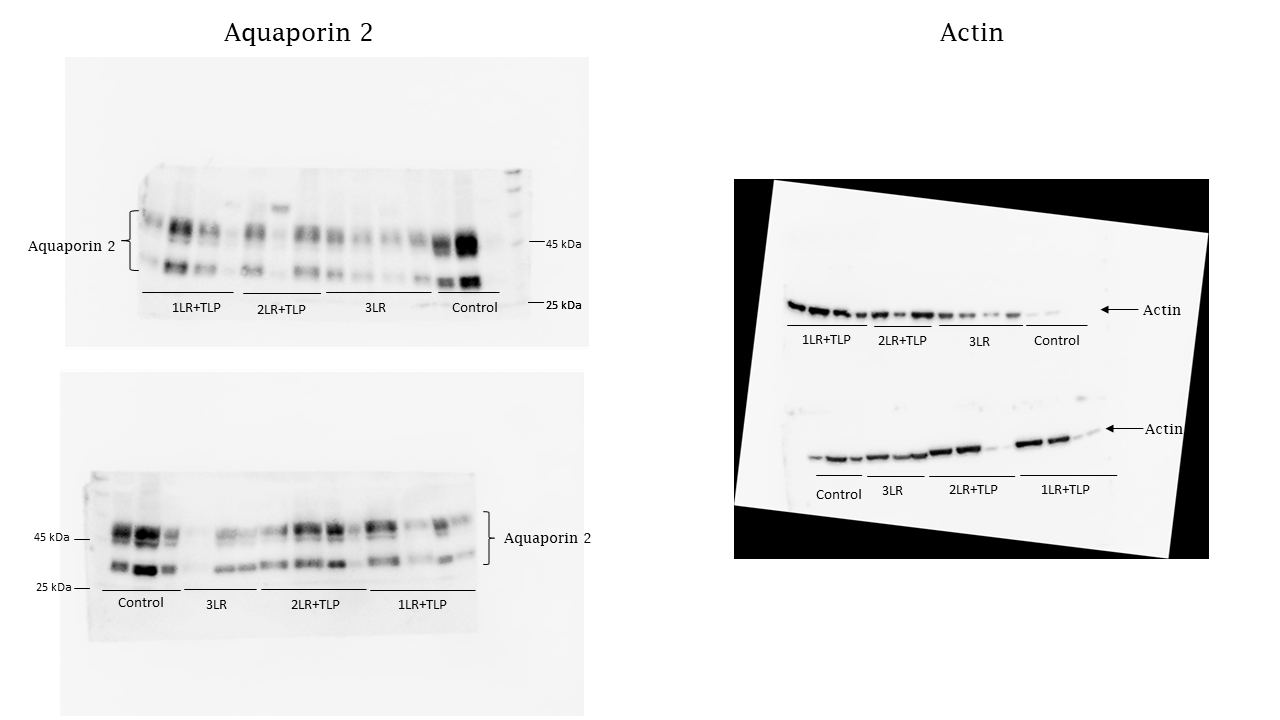

Supplement: Supplementary file 1 — Supplementary Figure S1. [file 41598_2022_24982_MOESM1_ESM.tif]

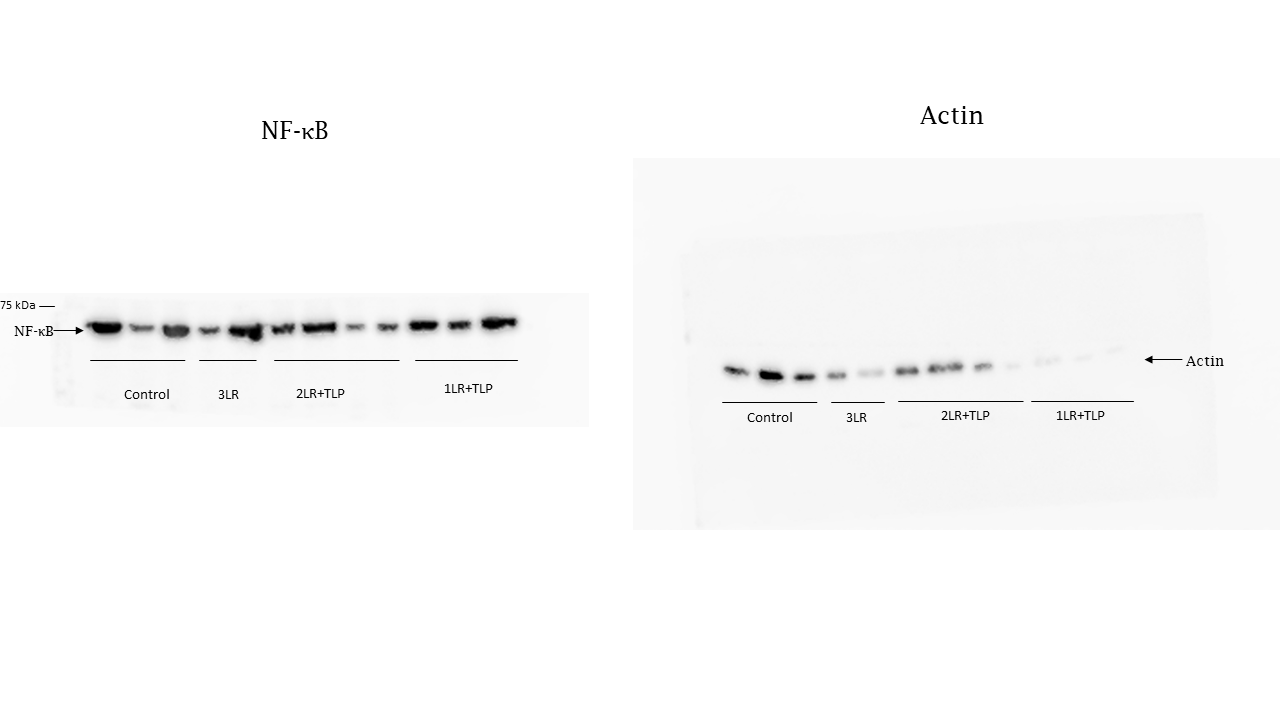

Supplement: Supplementary file 2 — Supplementary Figure S2. [file 41598_2022_24982_MOESM2_ESM.tif]

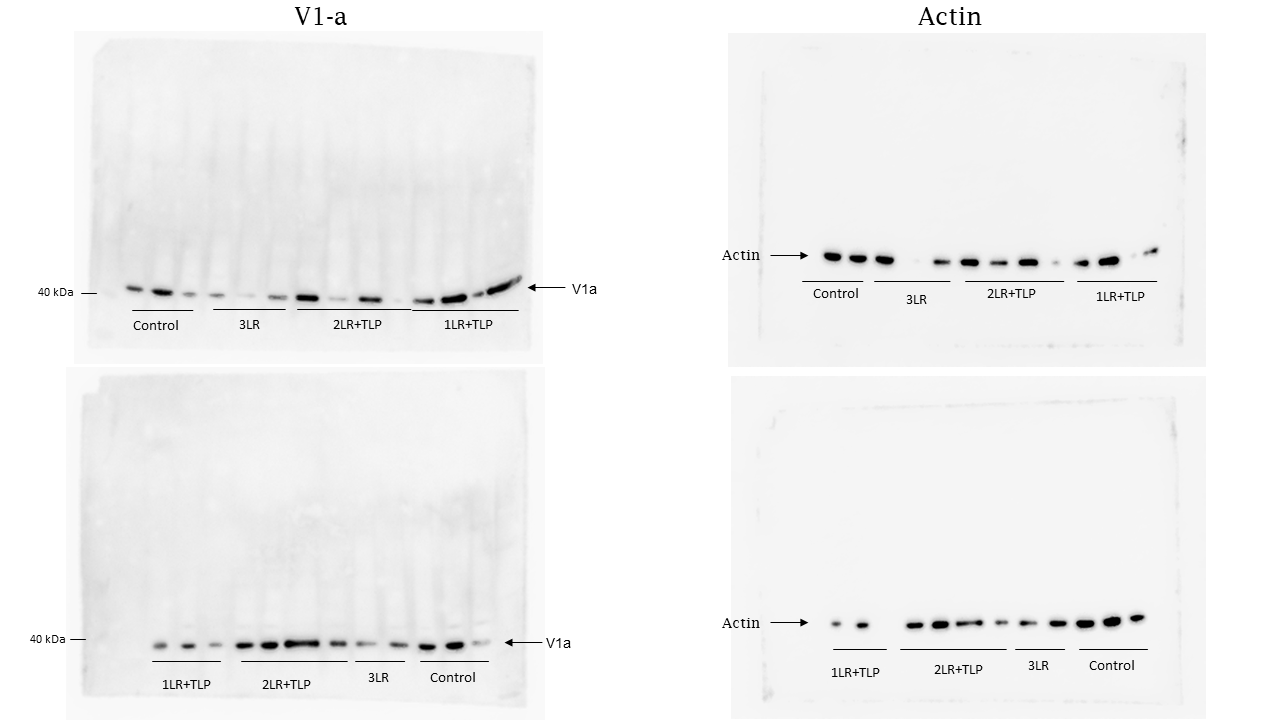

Supplement: Supplementary file 3 — Supplementary Figure S3. [file 41598_2022_24982_MOESM3_ESM.tif]
